# A *KRT71* Loss-of-Function Variant Results in Inner Root Sheath Dysplasia and Recessive Congenital Hypotrichosis of Hereford Cattle

**DOI:** 10.3390/genes12071038

**Published:** 2021-07-04

**Authors:** Joana G. P. Jacinto, Alysta D. Markey, Inês M. B. Veiga, Julia M. Paris, Monika Welle, Jonathan E. Beever, Cord Drögemüller

**Affiliations:** 1 Department of Veterinary Medical Sciences, University of Bologna, 40064 Ozzano Emilia, Italy; joana.goncalves2@studio.unibo.it; 2 Institute of Genetics, Vetsuisse Faculty, University of Bern, 3012 Bern, Switzerland; julia-1991@hotmail.com; 3 Laboratory of Molecular Genetics, Department of Animal Sciences, University of Illinois at Urbana-Champaign, Urbana, IL 61801, USA; alysta.markey@gmail.com (A.D.M.); jbeever@utk.edu (J.E.B.); 4 Institute of Animal Pathology, Vetsuisse Faculty, University of Bern, 3012 Bern, Switzerland; ines.veiga@vetsuisse.unibe.ch (I.M.B.V.); monika.welle@vetsuisse.unibe.ch (M.W.); 5 UTIA Genomics Center for the Advancement of Agriculture, Institute of Agriculture, University of Tennessee, Knoxville, TN 37996, USA

**Keywords:** *Bos taurus*, congenital hypotrichosis, hair, head domain, keratin 71, precision medicine

## Abstract

Genodermatoses, such as heritable skin disorders, mostly represent Mendelian conditions. Congenital hypotrichosis (HY) characterize a condition of being born with less hair than normal. The purpose of this study was to characterize the clinicopathological phenotype of a breed-specific non-syndromic form of HY in Hereford cattle and to identify the causative genetic variant for this recessive disorder. Affected calves showed a very short, fine, wooly, kinky and curly coat over all parts of the body, with a major expression in the ears, the inner part of the limbs, and in the thoracic-abdominal region. Histopathology showed a severely altered morphology of the inner root sheath (IRS) of the hair follicle with abnormal Huxley and Henle’s layers and severely dysplastic hair shafts. A genome-wide association study revealed an association signal on chromosome 5. Homozygosity mapping in a subset of cases refined the HY locus to a 690 kb critical interval encompassing a cluster of type II keratin encoding genes. Protein-coding exons of six positional candidate genes with known hair or hair follicle function were re-sequenced. This revealed a protein-changing variant in the *KRT71* gene that encodes a type II keratin specifically expressed in the IRS of the hair follicle (c.281delTGTGCCCA; p.Met94AsnfsX14). Besides obvious phenocopies, a perfect concordance between the presence of this most likely pathogenic loss-of-function variant located in the head domain of KRT71 and the HY phenotype was found. This recessive *KRT71*-related form of hypotrichosis provides a novel large animal model for similar human conditions. The results have been incorporated in the Online Mendelian Inheritance in Animals (OMIA) database (OMIA 002114-9913).

## 1. Introduction

Hair is one of the distinguishing characteristics of mammals, and is involved in a wide range of functions such as thermoregulation, physical protection, and sensory activity [1]. The hair follicle (HF) is responsible for the production of hair [2,3,4]. Moreover, the HF represents an ectodermal appendage of the skin and is a complex structure [5]. The growing hair shaft is molded by the inner root sheath (IRS), which is surrounded by the companion layer, the outer root sheath, and the fibrous sheath. The IRS is composed of three layers: the IRS cuticle, the Huxley layer, and the Henle layer [6]. Many genes and signaling are known to be involved in HF development [1].

Keratins are the main structural component of the HF and some epithelial keratins are highly specific for the IRS of the HF, such as a set of four type I keratins (KRT25-KRT28) and five type II keratins (KRT71-KRT75) [7]. In particular, KRT71 is known to play a major role in hair shaft molding [6]. All keratins belong to the family of intermediate filament (IF) proteins and therefore share a common structural organization composed of three domains: the N-terminal head domain; the central α-helical rod domain; and the C-terminal tail domain [8].

Heritable hair disorders similar to many other genodermatoses mostly follow a monogenic mode of inheritance, e.g., congenital hypotrichosis (HY). HY belong to a group of human diseases, which are largely classified into syndromic and non-syndromic forms. HY is a condition generally characterized by the presence of less than the normal amount of hair and abnormal hair follicles and shafts, which can be completely absent or are dysplastic. The extent of body hair involvement can be very variable [6]. Thus, HY encompasses a clinically- pathologically- and heritably-heterogeneous group of hair disorders. Currently, human non-syndromic HY classification distinguishes 14 subtypes and 12 different associated genes (OMIM PS605389). Human HY following a dominant inheritance have been associated with causative variants in eight different genes (*EPS8L3*, *SNRPE*, *CDSN*, *HR*, *KRT71*, *KRT74*, *RPL21*, *APCDD1*) [6,9,10,11,12,13,14,15]. Whereas, autosomal recessive HY is related to mutations in four different genes (*LIPH*, *LPAR6*, *DSG4*, *LSS*) [16,17,18,19].

Forms of non-syndromic HY have been reported in many animal species (OMIA 000540), including American minks [20], cats [21,22], dogs [23], horses [24], macaques [25], meadow voles [26], Mongolian gerbils [27], golden hamsters [28], guinea pigs [29], pigs [30], sheep [31] and cattle [32]. Pathogenic variants causing forms of HY in animals have been identified in known candidate genes for HY (*HR* and *KRT71*) [21,22], or novel genes (*TSR2*, *SGK3* and *SP6*) [23,24,32] in HY-affected domestic animals. This highlights the potential of studying inherited conditions in such species to assign a role or function to previously uncharacterized genes or to add additional functions to known genes in regard to hair development.

In Hereford cattle, the occurrence of HY has been previously reported [33]. Hereford animals affected with the HY phenotype are born with partial or complete absence of hair that later becomes “fuzzy or kinky” in appearance [33]. This Hereford phenotype appears to be limited to the hair coat with microscopic analysis showing abnormal hair development. Characteristic large, atypical trichohyalin granules form in the IRS and are associated with premature breakup of the sheath and loss of the hair [34,35,36]. Furthermore, pedigree analysis indicates an autosomal recessive mode of inheritance [33,35]. Therefore, a monogenic cause for this breed-specific form of bovine HY affecting a functional candidate gene was hypothesized.

The aim of this study was to characterize the clinical and histopathological phenotypes of HY in Hereford cattle and to map the responsible genetic locus in the bovine genome and to identify the causative genetic variant associated with the disorder.

## 2. Materials and Methods

### 2.1. Animals and Samples

Clinical and pathological investigations were performed in 2021 at the University of Bern using two affected animals (cases 1 and 2) noted at the same farm in Switzerland. In addition, blood samples of 31 unaffected animals including both dams were sampled at this farm for subsequent genotyping.

Genetic investigation that leads to the identification of the pathogenic variant associated with the disease was performed earlier at the University of Illinois. The mapping population in this study consisted of 17 suspected affected calves born in the USA reported by eleven different farmers between 2007 and 2010, with eight of those having a single common male ancestor suspected to be a carrier of hypotrichosis (cases 24, 21, 111, 70, 109, 105, 107 and 110; group A). In addition, 22 suspected carrier animals were collected from these farms. The remaining affected calves were from two separate groups, with the first being comprised of three affected half siblings and two distantly related calves (cases 22, 27, 341, 68, 237; group B). The second group contained four affected calves from three herds with unknown relatedness (cases 101, 287, 23, 286; group C). Finally, a population cohort consisting of 174 apparently normal cattle of the US purebred Hereford population was used to determine the absence/presence and frequency of the detected *KRT71* variant in the breed.

DNA was isolated from EDTA blood and semen samples using a simple salting out procedure [37].

### 2.2. Clinical and Histopathological Investigations

Two Hereford calves born in Switzerland, a two-month-old male (case 1) and an 11-month-old female (case 2), were referred by a breeder to the Institute of Genetics at the University of Bern because of congenital abnormal hair coat. The farm was a cow-calf-operation of Hereford cattle utilizing natural mating. Both cases were offspring from the same sire and both dams had a common ancestor. The two animals were reported to be born almost hairless and then started to develop short hair with age. The breeder reported that in the last four years, there were three additional cases in his herd.

Both affected calves were clinically examined. Furthermore, skin biopsies using an 8 mm punch were obtained from the neck skin from case 1 and a healthy control (1.5-month-old Simmental calf). The collected samples were fixed in 10% neutral buffered formalin and submitted to the Institute of Animal Pathology at the University of Bern, where these were trimmed, processed, embedded in paraffin wax, sectioned at 4 µm, and stained with hematoxylin and eosin (H&E) for further histological evaluation.

### 2.3. Genetic Investigations

#### 2.3.1. SNP Genotyping and GWAS

Seventeen affected animals and 54 normal breed controls were genotyped using the BovineSNP50 v1 Beadchip (Illumina, San Diego, CA, USA). 

The whole genome association study (GWAS) and homozygosity mapping were completed using PLINK [38]. The “--assoc” and “--mperm” commands were used for GWAS and to obtain the corrected empirical P-value (max(T)) with 10,000 permutations. The commands “--homozyg-group” and “--homozyg-verbose” were used to group the pools of overlapping segments and display the genotypes for each pool. The command “--mind 0.15” allowed 15% missing genotypes per individual while the command “--maf 0.01” set the minor allele frequency to 1%. The command “--cow” set the chromosome codes for the cow. The command “--allow-no-sex” was used to allow ambiguously sexed individuals. The command “--homozyg-density 100” was used to allow one SNP per 100 kb.

During the genomic analysis, three different groups of affected animals were considered: group (A) represented eight cases that appeared in the verbose file using the homozygosity analysis commands described in Section 2.1; group (B) represents five cases that also appeared in the verbose file using relaxed homozygosity analysis commands; and group (C) represents four cases that did not appear in a verbose file from homozygosity analysis.

#### 2.3.2. Microsatellite Genotyping

Microsatellite mapping was completed with 17 affected and 22 suspected carrier animals. Microsatellites were selected with the simple sequence repeat identification tool (SSRIT) [39], sequences were masked using RepeatMasker [40] and primers were designed using Primer Designer v 2.0 (Scientific and Educational Software). PCR products were fluorescently tagged via an M13 protocol [41], amplified in multiplex PCR and fragment analysis performed using an ABI3730xl DNA Analyzer (Applied Biosystems, Foster City, CA, USA). Microsatellite genotypes were analyzed with GeneMarker™ (Softgenetics^®^, LLC, State College, PA, USA).

#### 2.3.3. Candidate Gene Analysis, Targeted Genotyping, Occurrence of the KRT71 Variant in a Global Control Cohort

Resequencing of candidate genes was completed for two normal, three suspected carrier and three affected animals. Six keratin genes (*KRT72*, *KRT71*, *KRT74*, *KRT83*, *KRT86*, and *KRT81*) were selected as candidates based on known function in hair and location relative to the homozygous region identified in the HY-affected calves on chromosome 5. Exon annotation based on computational methods was manually validated using mRNA sequences from NCBI and the software SPIDEY [42]. All references to the bovine *KRT71* gene correspond to the NCBI accessions NM_001075970.1 (*KRT71* mRNA), NP_001069438.1 (KRT71 protein), NC_037332.1 (ARS-UCD1.2 assembly, chromosome 5). For the protein structure of KRT71 the Uniprot accession Q148H5 was used.

Primers were designed as describe above to amplify protein coding exons in 1kb fragments (Table S1). Subsequent Sanger sequencing was performed on an ABI3730xl capillary sequencer and sequence assemblies were viewed and analyzed for polymorphisms using a Codon Code Aligner (Codon Code Corporation).

A diagnostic PCR and subsequent Sanger sequencing as described above were used to validate and genotype the variant in further animals. Therefore, the region containing the 8-bp deletion in *KRT71* (g.27331221delTGTGCCCA) was amplified using the following primers: 5′-CAGTGGGAAGAGTGGAGGTT-3′ (forward primer) and 5′-CAATCCCTCTTGCTGCAACA-3′ (reverse primer).

The most likely pathogenic 8-bp deletion in *KRT71* was searched for its occurrence in a global control cohort of 4110 genomes of a variety of breeds (1000 Bull Genomes Project run 8; www.1000bullgenomes.com (accessed on 1 July 2021)) [43]. Therefore we inspected the provided Variant Call Format (VCF) file of bovine chromosome 5 (PRJEB42783 is the project accession number at the European Variation Archive; http://www.ebi.ac.uk/en (accessed on 1 July 2021)) to evaluate the genotypes of the g.27331221delTGTGCCCA variant using an awk command on a local Linux server at the University of Bern.

## 3. Results

### 3.1. Clinical Phenotype

Particularly, the clinical examination of the cardiovascular, respiratory, urinary, musculoskeletal, and nervous systems showed no abnormalities. Moreover, no abnormalities in dentition were noticed as previously seen in cattle affected by ectodermal dysplasia, which is characterized by sparse hair and abnormal teeth [44]. 

**Figure 1:**
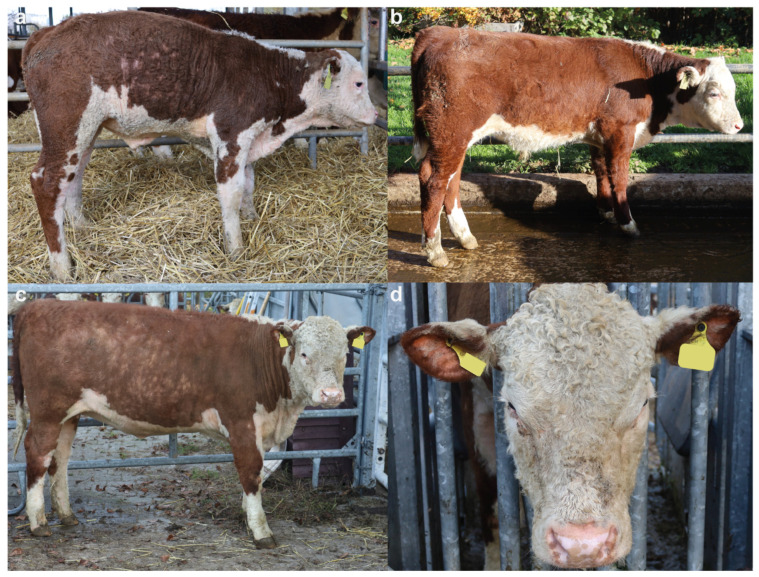
Congenital hypotrichosis in Hereford cattle. (**a**) Case 1: note the short, fine, wooly, kinky and curly hair, particularly in the thoracic-abdominal region. (**b**) Healthy control: 2-month-old Hereford calf with a normal coat. (**c**) Case 2: note the short, fine, wooly, kinky and curly hair, which is more severe than in case 1. (**d**) Case 2: note the evident curly hair in the head and the short, fine and kinky hair in the ears.

The examination of the integument of both calves showed very short, fine, wooly, kinky and curly hair when compared to healthy animals (Figure 1a–c). The curly hair was more prominent in case 2, mostly on the head (Figure 1d). Kinky hair appeared over all parts of the body, with a major expression in ears, inner part of the limbs, and in the thoracic-abdominal region (Figure 1a,c,d). In the older animal (case 2), the observed lesions were more obvious (Figure 1c,d). No ectoparasites were observed in either case. Clinically, the calves were found to be healthy, with the exception of the hair coat changes.

Based on these clinical observations, the calves were suspected to have a congenital hypotrichosis.

### 3.2. Histopathological Phenotype

**Figure 2:**
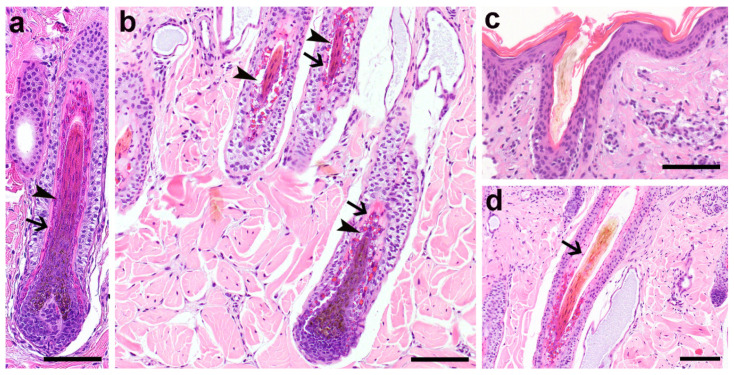
Histological changes displayed by a Hereford calf with congenital hypotrichosis. (**a**) Proximal portion of an anagen HF of a 1,5-month-old Simmental calf with a normal, non-cornified Huxley layer (arrowheads) and an already cornified Henle layer of the IRS (arrows) for comparison purposes. H&E staining, 200×. (**b**) Proximal portion of the anagen hair follicles of an affected Hereford calf (case 1) with a severely altered morphology of the inner root sheath. The cells of the non-cornified Huxley layer present with large, irregular sized trichohyalin granules and large vacuoles (arrowheads) in the cytoplasm. The already cornified Henle’s layer (arrows) presents with large corneocytes with irregular staining characteristics. H&E staining, 200×. (**c**) Infundibulum with a dysplastic hair shaft from case 1, characterized by an irregular outer contour. H&E staining, 200×. (**d**) Altered cornification of the IRS in the isthmus region in case 1. The cornified IRS appears wider than normal and displays irregular staining characteristics. Nuclei were not arranged orderly (arrow) and the hair shaft has an irregular contour. H&E staining, 200×, bar 100 µm in (**a**,**b**,**d**); bar 50 µm in (**c**).

Histologically, the proximal portions of the hair follicles presented with dysplastic changes of the inner root sheath, characterized by large and irregularly sized trichohyalin granules and vacuolated cytoplasm of the non-cornifed IRS cells (Figure 2b). The cornified IRS of the Henley layer in the inferior portion and of the Henley and Huxley layer of the isthmic region displayed irregular staining characteristics, and the remaining nuclei present in the isthmus were distributed irregularly (Figure 2b,d), whereas in normal bovine skin they are equally distributed (Figure 2a). Hair shafts were often shaped irregularly with an altered outer contour and were occasionally thinner than normal (Figure 2c,d). The histological findings were compatible with the clinical suspicion of a congenital hypotrichosis.

### 3.3. Genetic Analysis

**Figure 3:**
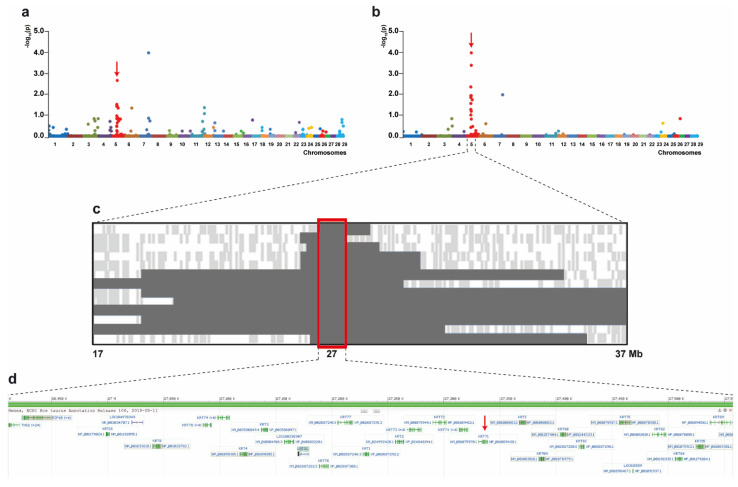
Positional cloning of the locus for recessive congenital hypotrichosis (HY) in Hereford cattle. (**a**) GWAS case/control results considering all purported HY-suspicious calves. Each chromosome is color coded along the X axis. The Y axis represents the –Log_10_ of the corrected empirical P-value (max(T)) after 10,000 permutations from PLINK. (**b**) GWAS case/control results excluding calves from group C. Each chromosome is color coded along the X axis. The Y axis represents the –Log_10_ of the corrected empirical P-value (max(T)) after 10,000 permutations from PLINK. (**c**) Schematic representing the genotypes of 13 HY-affected calves on chromosome 5. Each horizontal lane represents one calf with dark grey shading, indicating shared homozygosity. Light grey shading indicates a heterozygous genotype and white indicates an alternative homozygous genotype. The approximate Mb position is indicated below the figure. The red box encompasses the consensus homozygous region which spans approximately 690 kb. (**d**) Gene content of the critical region. Screen shot of NCBI Genome Data Viewer (ARS-UCD1.2 assembly, *Bos taurus* annotation release 106) shows the cluster of keratin II encoding genes including *KRT71* (red arrow).

Based on pedigree records, a monogenic recessive mode of inheritance was hypothesized. Initial GWAS using all 17 cases and 54 controls revealed evidence for association with the HY phenotype on chromosomes 5 and 7 (Figure 3a). Subsequent manual inspection of SNP genotyping data in both genome regions revealed a 690 kb region of shared homozygosity only for chromosome 5 from 26.91 Mb to 27.60 Mb in thirteen affected calves of group A and B (Figure 3c) while microsatellite genotyping of all 13 cases confirmed the region as homozygous between 26.58 Mb and 27.56 Mb (Table S2). The four HY-suspicious calves (group C) were excluded from this inspection, as they were heterozygous at different markers flanking this region (Table S2). Subsequent removal of these four calves from GWAS resulted in greater statistical significance of the association on chromosome 5 (Figure 3b) when compared to the GWAS including all reported calves (Figure 3a).

**Figure 4:**
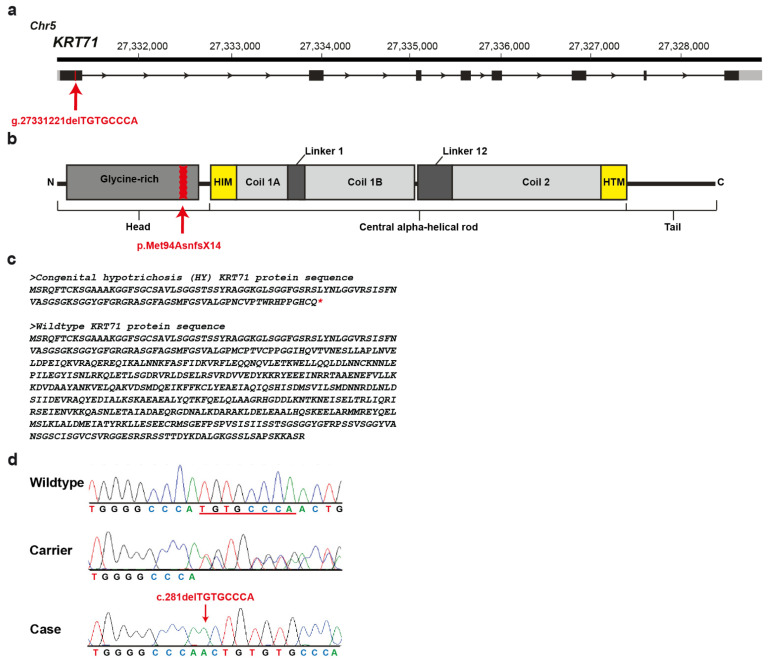
*KRT71* deletion in Hereford cattle affected by congenital hypotrichosis. (**a**) *KRT71* gene structure showing the location of the exon 1 variant on cattle chromosome 5. (**b**) Schematic representation of KRT71 protein with its three functional domains. (**c**) Predicted protein sequences of an HY and wildtype KRT71 protein. The red star (*) represents a stop codon. The normal protein is 525 amino acid residues in length. The HY protein is expected to be 108 amino acid residues in length due to the premature induction of a stop codon. (**d**) Electropherograms showing the different genotypes identified via Sanger sequencing.

Positional candidate genes within the critical region were selected based on function and location relative to the homozygosity analysis of the HY-affected calves. A cluster of type II keratin encoding genes is annotated in that region (Figure 3d) and a total of 40 protein coding exons of six positional candidate genes were re-sequenced: *KRT72*, *KRT71*, *KRT74*, *KRT83*, *KRT86*, and *KRT81*. Only variants that were predicted to alter the coding sequences or that were located within the splice sites were considered. An eight base pair deletion was found in the first exon of the *KRT71* gene (chr5: g.27331221delTGTGCCCA) (Figure 4a), which was consistent with expected genotype status of the animals sequenced. At the level of translation, the detected deletion (c.281delTGTGCCCA) is predicted to result in a frameshift in the head domain of KRT71 after methionine 94 with a premature stop codon at threonine 108 (p.Met94AsnfsX14) (Figure 4b,c). Consequently, the mutant protein, if expressed, is predicted to be significantly shorter than the normal KRT71 protein of 525 amino acids in length lacking the central α-helical rod and the terminal tail-domains important for dimerization (Figure 4c).

The 8-bp frameshift deletion in *KRT71* was validated by Sanger sequencing (Figure 4d). Genotyping of all 72 animals of the mapping cohort including the HY-suspicious calves, carriers and controls originating from the US Hereford population plus the cases, carriers and controls of the affected herd from Switzerland revealed perfect concordance between the presence of this deletion and the HY phenotype (Table 1). Among the HY- cases, all but four from the mapping cohort were homozygous mutants and all available carriers were heterozygous. These four HY-suspicious calves (case 110 of group A and cases 287, 23, 286 of group C) did not show the deleterious variant in *KRT71* nor a protein-changing variant in any further exon of the six candidate genes that were re-sequenced. It is probable that these four calves were misclassified based on phenotype and were not affected with the same type of congenital hypotrichosis and therefore were considered to represent phenocopies. Calf 101 (group C) was homozygous for the 8-bp frameshift deletion in *KRT71* but showed a shorter region of homozygosity, limiting the critical interval to 301 kb. Altogether, the mutant allele frequency was estimated as 2.2% in the studied unrelated 235 normal Hereford cattle.

Additionally, the identified *KRT71* variant was absent in a global control cohort of 4110 cattle genomes, including 117 Hereford animals, of a variety of breeds included in the run 8 of the 1000 Bull Genomes Project [43].

## 4. Discussion

The identified deletion in *KRT71* resulting in a frameshift and early truncation of the protein affects a functionally important site of an obvious candidate gene and thus represents the most likely pathogenic variant associated with the observed recessively inherited congenital hypotrichosis (HY) phenotype in Hereford cattle. The HY-affected Hereford cattle herein presented clinically showed short, fine, wooly, kinky and curly hair and the skin biopsies examined from one of the affected calves showed a severely altered morphology of the IRS and the hair shafts.

Type I keratins have an acidic charge and are generally smaller while type II keratins, including *KRT71*, have a basic charge and are larger. These keratins form obligate type I-type II heterodimers [7,8]. Particularly, cytoplasmic IF proteins, such as keratins, undergo post-translational modifications (PMTs), including phosphorylation, glycosylation, sumoylation, acetylation, prenylation, ubiquitylation and transamidation, which regulate each other through crosstalk and binding of IFs to other proteins [45]. The structure of these proteins is highly dynamic and is able to reorganize in specific physiological conditions, e.g., during mitosis, cell stress situations and mutation response. The specific site of phosphorylation in the head and tail residues is the main facilitator of IF reorganization. Additionally, IF undergo other PMTs with target residues seated in the head, tail and central α-helical rod domains. PMTs of the central α-helical rod domain are most likely facilitated by the head and tail domain. It is known that many PMTs are altered in the context of disease-causing IF mutant [45]. The mutation identified in this study was positioned in the head domain of the protein. Taking into account that the identified pathogenic variant is a frameshift deletion resulting in a significantly shorter mutant transcript, and since this domain plays an essential role in the regulation of the protein, we speculate that the identified deletion disturbs the function. In fact, if the mutant mRNA transcript were to escape nonsense-mediated decay [46], the resultant truncated protein would not contain the α-helical rod domain or the helix boundary motifs that are integral to heterodimer formation of keratin molecules. This truncated protein would likely be unable to participate in filament formation, resulting in instability in the cytoskeletal network and a lack of tissue integrity [47].

So far, *KRT71* variants have been identified and studied at a molecular level in humans (OMIM 608245), mice, rats, dogs (OMIA 000245-9615) and cats (OMIA 001581-9685, OMIA 001712-9685, OMIA 001583-9685). In veterinary medicine, just one autosomal recessive splice site variant in *KRT71*-related hypotrichosis has been reported in cats in the coil 2 region of the central α-helical rod domain [22] and the histological findings in the hair follicles of Sphinx cats, which are a hairless cat breed, are identical to those found in the presented case [48]. In human medicine, a single, dominantly inherited *KRT71* missense variant associated with hyptrichosis has been reported affecting the helix initiation motif of the central α-helical rod domain [6]. Moreover, in mice models, several pathogenic *Krt71* variants affecting a single amino acid placed in the α-helical rod domain, mostly following an autosomal dominant inheritance, have been reported [49,50]. Interestingly, in mice, just one variant was positioned in the head domain and also showed recessive inheritance (*Rco3* mutation) [51]. The *Rco3* mutation is a 10 base pair deletion, resulting in a frameshift after amino acid residue 58 and, therefore, the absence of 422 carboxyl-terminal amino acid residues containing the complete α-helical rod domain [51]. The amino acid residues 59–134 show no similarity to any known or predicted protein. Similarly, the herein identified deletion is predicted to result in truncation of the KRT71 protein after amino acid residue 108 with residues 95–108 showing no similarity to keratin proteins. Both truncated proteins would lack the rod and tail domains as well as the helix boundary motifs that are critical for heterodimer formation. Thus, if a truncated KRT71 protein is produced, it is unlikely that the mutant protein would participate in correct heterodimer formation. As there were no detectable levels of KRT71 in the IRS of the *Rco3* mice, it is more likely that the aberrant bovine KRT71 transcript was removed by nonsense-mediated decay [46,51]. The same can be assumed for the variant identified here in cattle.

Further similarities between the *Rco3* and bovine variants are seen comparing the HY-phenotypes. The *Rco3* mutation is also autosomal and is recessively inherited and affects the skin, coat and nails, as well as the touch and vibrissae systems. The first body coat of *Rco3* homozygous mice was curly in nature, while the second body coat displayed progressive alopecia that was severe and patchy. Hair texture was abnormal with the cortex displaying frequent kinks and twists. The homozygous *Rco3* mice showed defective keratinization of the Henle’s and Huxley’s layers of the IRS, similar to what was observed in HY-affected Hereford cattle. Furthermore, the Henle’s layer of *Rco3* mutants displayed accumulation of electron-dense material, as well as lack of normal filament bundles that is likely due to either the inability of the truncated or the lack of the entire KRT71 protein to form heterodimers and intermediate filaments [51]. However, as described before in Sphinx and Devon Rex cats, also in the herein presented Hereford cattle, nails (respective hooves) and vibrissae system abnormalities as shown in *Rco3* mice were absent.

The hair anomalies reported here in Hereford cattle differ from those recently described in *HEPHL1*-related HY of Galloway cattle [52].

## 5. Conclusions

Rare non-lethal disorders such as hypotrichosis in livestock are usually not reported or diagnosed when the animals show mild to moderate phenotype, but they affect animal welfare through secondary wounds and may result in UV-induced skin cancer in grazing animals, thus lower the value of the affected animals. Additionally, molecular diagnosis is often not performed because of a lack of resources and diagnostic tools. Furthermore, this study provides a DNA-based diagnostic test that allows selection against the identified pathogenic variant in the international Hereford population. Investigation of these cases allowed a clinical, histopathological, and molecular genetic study, enabling for the first time the diagnosis of a *KRT71*-related recessively inherited form of HY in Hereford cattle. The loss-of-function variant most likely results to nonoccurrence of KRT71 during hair shaft molding, explaining the hair disorder. This study represents the first large animal model for similar human conditions. This example highlights the utility of precision diagnostics for understanding rare disorders and the neglected value of livestock populations for studying genetic disorders.

## Figures and Tables

**Table 1:** Association of the 8-bp deletion with the hypotrichosis phenotype.

	wt/wt	wt/del	del/del
HY-affected calves			
Swiss cases ^b^			2
US cases	4 ^a^		13
Obligate carriers ^c^			
Swiss ^b^		2	
US		22	
Unrelated normal Hereford cattle			
Swiss ^b^	21	8	
US	197	9	
Global cohort included in the 1000 Bull Genomes Project	117		
Normal control cattle from various different breeds	3993		

^a^ these animals were assumed to represent phenocopies (see main text). ^b^ these animals were collected in a single farm. ^c^ parents of affected animals were classified as obligate carriers.

## Data Availability

Not applicable.

## Supplementary Materials

The following are available online at https://www.mdpi.com/article/10.3390/genes12071038/s1. Table S1: Primer Sequences. Table S2. Genotypes of reported HY-suspicious calves on chromosome 5.

## References

[1] Schneider M.R., Schmidt-Ullrich R., Paus R. (2009). The Hair Follicle as a Dynamic Miniorgan. Curr. Biol..

[2] Aoki N., Sawada S., Rogers M.A., Schweizer J., Shimomura Y., Tsujimoto T., Ito K., Ito M. (2001). A novel type II cytokeratin, mK6irs, is expressed in the Huxley and Henle layers of the mouse inner root sheath. J. Investig. Dermatol..

[3] Porter R.M., Gandhi M., Wilson N.J., Wood P., McLean W.H.I., Lane E.B. (2004). Functional analysis of keratin components in the mouse hair follicle inner root sheath. Br. J. Dermatol..

[4] Higgins C.A., Westgate G.E., Jahoda C.A.B. (2011). Modulation in proteolytic activity is identified as a hallmark of exogen by transcriptional profiling of hair follicles. J. Investig. Dermatol..

[5] Shimomura Y., Christiano A.M. (2010). Biology and genetics of hair. Annu. Rev. Genom. Hum. Genet..

[6] Fujimoto A., Farooq M., Fujikawa H., Inoue A., Ohyama M., Ehama R., Nakanishi J., Hagihara M., Iwabuchi T., Aoki J. (2012). A missense mutation within the helix initiation motif of the keratin K71 gene underlies autosomal dominant woolly hair/hypotrichosis. J. Investig. Dermatol..

[7] Moll R., Divo M., Langbein L. (2008). The human keratins: Biology and pathology. Histochem. Cell Biol..

[8] Coulombe P.A., Omary M.B. (2002). “Hard” and “soft” principles defining the structure, function and regulation of keratin intermediate filaments. Curr. Opin. Cell Biol..

[9] Zhang X., Guo B.R., Cai L.Q., Jiang T., Sun L.D., Cui Y., Hu J.C., Zhu J., Chen G., Tang X.F. (2012). Exome sequencing identified a missense mutation of EPS8L3 in Marie Unna hereditary hypotrichosis. J. Med. Genet..

[10] Pasternack S.M., Refke M., Paknia E., Hennies H.C., Franz T., Schäfer N., Fryer A., Van Steensel M., Sweeney E., Just M. (2013). Mutations in SNRPE, which encodes a core protein of the spliceosome, cause autosomal-dominant hypotrichosis simplex. Am. J. Hum. Genet..

[11] Betz R.C., Lee Y.A., Bygum A., Brandrup F., Bernal A.I., Toribio J., Alvarez J.I., Kukuk G.M., Ibsen H.H.W., Rasmussen H.B. (2000). A gene for hypotrichosis simplex of the scalp maps to chromosome 6p21.3. Am. J. Hum. Genet..

[12] Kim J.K., Kim E., Baek I.C., Kim B.K., Cho A.R., Kim T.Y., Song C.W., Seong J.K., Yoon J.B., Stenn K.S. (2009). Overexpression of Hr links excessive induction of Wnt signaling to Marie Unna hereditary hypotrichosis. Hum. Mol. Genet..

[13] Wasif N., Naqvi S.K.U.H., Basit S., Ali N., Ansar M., Ahmad W. (2011). Novel mutations in the keratin-74 (KRT74) gene underlie autosomal dominant woolly hair/hypotrichosis in Pakistani families. Hum. Genet..

[14] Zhou C., Zang D., Jin Y., Wu H., Liu Z., Du J., Zhang J. (2011). Mutation in ribosomal protein L21 underlies hereditary hypotrichosis simplex. Hum. Mutat..

[15] Shimomura Y., Agalliu D., Vonica A., Luria V., Wajid M., Baumer A., Belli S., Petukhova L., Schinzel A., Brivanlou A.H. (2010). APCDD1 is a novel Wnt inhibitor mutated in hereditary hypotrichosis simplex. Nature.

[16] Kazantseva A., Goltsov A., Zinchenko R., Grigorenko A.P., Abrukova A.V., Moliaka Y.K., Kirillov A.G., Guo Z., Lyle S. (2006). Human hair growth deficiency is linked to a genetic defect in the phospholipase gene LIPH. Science.

[17] Pasternack S.M., Von Kügelgen I., Al Aboud K., Lee Y.-A., Rüschendorf F., Voss K., Hillmer A.M., Molderings G.J., Franz T., Ramirez A. (2008). G protein-coupled receptor P2Y5 and its ligand LPA are involved in maintenance of human hair growth. Nat. Genet..

[18] Shimomura Y., Sakamoto F., Kariya N., Matsunaga K., Ito M. (2006). Mutations in the desmoglein 4 gene are associated with monilethrix-like congenital hypotrichosis. J. Investig. Dermatol..

[19] Romano M.T., Tafazzoli A., Mattern M., Sivalingam S., Wolf S., Rupp A., Thiele H., Altmüller J., Nürnberg P., Ellwanger J. (2018). Bi-allelic mutations in LSS, encoding lanosterol synthase, cause autosomal-recessive hypotrichosis simplex. Am. J. Hum. Genet..

[20] Johansson I. (1965). Congenital defects in mink. Vara Palsdjur..

[21] Buckley R.M., Gandolfi B., Creighton E.K., Pyne C.A., Bouhan D.M., Leroy M.L., Senter D.A., Gobble J.R., Abitbol M., Lyons L.A. (2020). Werewolf, there wolf: Variants in hairless associated with hypotrichia and roaning in the lykoi cat breed. Genes.

[22] Gandolfi B., Outerbridge C.A., Beresford L.G., Myers J.A., Pimentel M., Alhaddad H., Grahn J.C., Grahn R.A., Lyons L.A. (2010). The naked truth: Sphynx and Devon Rex cat breed mutations in KRT71. Mamm. Genome.

[23] Parker H.G., Harris A., Dreger D.L., Davis B.W., Ostrander E.A. (2017). The bald and the beautiful: Hairlessness in domestic dog breeds. Philos. Trans. R. Soc. B Biol. Sci..

[24] Thomer A., Gottschalk M., Christmann A., Naccache F., Jung K., Hewicker-Trautwein M., Distl O., Metzger J. (2018). An epistatic effect of KRT25 on SP6 is involved in curly coat in horses. Sci. Rep..

[25] Ratterree M.S., Baskin G.B. (1992). Congenital hypotrichosis in a rhesus monkey. Lab. Anim. Sci..

[26] Pinter A.J., McLean A.K. (1970). Hereditary hairlessness in the montane vole. J. Hered..

[27] Swanson H. (1980). The “hairless” gerbil: A new mutant. Lab. Anim..

[28] Nixon C. (1972). Hereditary hairlessness in the Syrian golden hamster. J. Hered..

[29] Bolognia J.L., Murray M.S., Pawelek J.M. (1990). Hairless pigmented guinea pigs: A new model for the study of mammalian pigmentation. Pigment Cell Res..

[30] Lemus-Flores C., Ulloa-Arvizu R., Ramos-Kuri M., Estrada F.J., Alonso R.A. (2001). Genetic analysis of Mexican hairless pig populations. J. Anim. Sci..

[31] Finocchiaro R., Portolano B., Damiani G., Caroli A., Budelli E., Bolla P., Pagnacco G. (2003). The hairless (hr) gene is involved in the congenital hypotrichosis of Valle del Belice sheep. Genet. Sel. Evol..

[32] Murgiano L., Shirokova V., Welle M.M., Jagannathan V., Plattet P., Oevermann A., Pienkowska-Schelling A., Gallo D., Gentile A., Mikkola M. (2015). Hairless streaks in cattle implicate TSR2 in early hair follicle formation. PLoS Genet..

[33] Craft W.A., Blizzard W.L. (1934). The inheritance of semi-hairless ness in cattle. J. Hered..

[34] Bracho G.A., Beeman K., Johnson J.L., Leipold H.W. (1984). Further studies of congenital hypotrichosis in Hereford cattle. Zent. Vet..

[35] Olson T.A., Hargrove D.D., Leipold H.W. (1985). Occurrence of Hypotrichosis in Polled Hereford Cattle. Bov. Pract..

[36] Jayasekara M.U., Leipold H.W., Cook J.E. (1979). Pathological changes in congenital hypotrichosis in Hereford cattle. Zentralbl. Veterinarmed. A.

[37] Miller S.A., Dykes D.D., Polesky H.F. (1988). A simple salting out procedure for extracting DNA from human nucleated cells. Nucleic Acids Res..

[38] Purcell S., Neale B., Todd-Brown K., Thomas L., Ferreira M.A.R., Bender D., Maller J., Sklar P., De Bakker P.I.W., Daly M.J. (2007). PLINK: A tool set for whole-genome association and population-based linkage analyses. Am. J. Hum. Genet..

[39] Temnykh S., DeClerck G., Lukashova A., Lipovich L., Cartinhour S., McCouch S. (2001). Computational and experimental analysis of microsatellites in rice (*Oryza sativa L.*): Frequency, length variation, transposon associations, and genetic marker potential. Genome Res..

[40] Saha S., Bridges S., Magbanua Z.V., Peterson D.G. (2008). Computational Approaches and Tools Used in Identification of Dispersed Repetitive DNA Sequences. Trop. Plant Biol..

[41] Boutin-Ganache I., Raposo M., Raymond M., Deschepper C.F. (2001). M13-tailed primers improve the readability and usability of microsatellite analyses performed with two different allele-sizing methods. Biotechniques.

[42] Wheelan S.J., Church D.M., Ostell J.M. (2001). Spidey: A tool for mRNA-to-genomic alignments. Genome Res..

[43] Hayes B.J., Daetwyler H.D. (2019). 1000 Bull Genomes Project to Map Simple and Complex Genetic Traits in Cattle: Applications and Outcomes. Annu. Rev. Anim. Biosci..

[44] O’Toole D., Häfliger I.M., Leuthard F., Schumaker B., Steadman L., Murphy B., Drögemüller C., Leeb T. (2021). X-linked hypohidrotic ectodermal dysplasia in crossbred beef cattle due to a large deletion in *EDA*. Animals.

[45] Snider N.T., Omary M.B. (2014). Post-translational modifications of intermediate filament proteins: Mechanisms and functions. Nat. Rev. Mol. Cell Biol..

[46] Arin M.J. (2009). The molecular basis of human keratin disorders. Hum. Genet..

[47] Langbein L., Rogers M.A., Praetzel S., Winter H., Schweizer J. (2003). K6irs1, K6irs2, K6irs3, and K6irs4 represent the inner-root-sheath-specific type II epithelial keratins of the human hair follicle. J. Investig. Dermatol..

[48] Genovese D.W., Johnson T.L., Lamb K.E., Gram W.D. (2014). Histological and dermatoscopic description of sphynx cat skin. Vet. Dermatol..

[49] Fairfield H., Srivastava A., Ananda G., Liu R., Kircher M., Lakshminarayana A., Harris B.S., Karst S.Y., Dionne L.A., Kane C.C. (2015). Exome sequencing reveals pathogenic mutations in 91 strains of mice with Mendelian disorders. Genome Res..

[50] Runkel F., Klaften M., Koch K., Böhnert V., Büssow H., Fuchs H., Franz T., De Angelis M.H. (2006). Morphologic and molecular characterization of two novel Krt71 (Krt2-6g) mutations: Krt71rco12 and Krt71rco13. Mamm. Genome.

[51] Peters T., Sedlmeier R., Büssow H., Runkel F., Lüers G.H., Korthaus D., Fuchs H., Hrabé De Angelis M., Stumm G., Russ A.P. (2003). Alopecia in a novel mouse model RCO3 is caused by mK6irs1 deficiency. J. Investig. Dermatol..

[52] Kuca T., Marron B.M., Jacinto J.G.P., Paris J.M., Gerspach C., Beever J.E., Drögemüller C. (2021). A nonsense variant in hephaestin like 1 (*HEPHL1*) is responsible for congenital hypotrichosis in Belted Galloway cattle. Genes..

